# 

**Published:** 1910-07

**Authors:** 


					﻿Sixty=fifth Year.
Vol. LXV.	JULY, 1 9/o. f 1 ft tli
buffaW-'
MEDICALJOURNAL
Established 1845 by AUSTIN FLINT M. D.
( lnck xe J
EDITOR:	v
WILLIAM WARREN POTTER, M. D.
ASSISTANT EDITORS:
NELSON W. WILSON, M. D.	JAMES WRIGHT PUTNAM, M. D,
I
ASSOCIATE EDITORS:
ERNEST WENDE, M. D. JOHN A. RAFTER, M. D.
JOHN PARMENTER, M. D.	JOHN A. MILLER, Ph. D.
MAUD J. FRYE, M. D.
				

